# 

**Published:** 1899-06-17

**Authors:** 


					THE HOSPITAL. "The standard of highest purity."-Lancet. J*"? 17th, 1899.
CADBURY'S U fc* CADBURY'S
fCL Ascan WrsTErw Inf'i'rmary
No. 664! Vol. XXVI. [jgg'4e*3 Saturday,
June 17th, 1899.
PRICE TWOPENCE.
see p. 192.

				

